# Skill acquisition in TVET and access to employment in Nigeria: a gender perspective

**DOI:** 10.3389/fsoc.2025.1577765

**Published:** 2025-04-23

**Authors:** Immaculata E. Emah, Philippe Doneys, Kyoko Kusakabe, Shubham Pathak

**Affiliations:** ^1^Asian Institute of Technology, Bangkok, Thailand; ^2^School of Accountancy and Finance, Center of Excellence in Sustainable Disaster Management (CESDM), Walailak University, Thai Buri, Thailand

**Keywords:** TVET (technical and vocational education and training), skill acquisition, gender, employment, graduates and the employment

## Abstract

**Introduction:**

Gender roles define women’s involvement in skilled labour, leaving women with low skills and income while responsible for devalued household work. This study sought to determine gender bias in skills acquired by TVET employees (graduates) and the impact of gender on employment opportunities of TVET graduates who are employees in TVET business organisations.

**Method:**

A construct of 5 hard skills and 7 soft skill sets was made from the literature to assess skill acquisition from employees in seven TVET fields: Business studies, radio, television, and electronics (RTE) repair, automobile vehicle repairs, block laying, bricklaying and concreting, electrical installation, hotel management and catering, and welding and fabrication.

**Results:**

Responses from a survey questionnaire and quantitative data analysis revealed no gender difference in the courses taken by TVET graduates (*χ*^2^ = 2.82; df = 6; *p* = 0.831). There was a gender bias (*p* < 0.05) in skills perceived to have been acquired by the TVET graduates. Gendered job descriptions defined the bias in skills such that women in hotel management/catering had the edge over men in all skills. In contrast, men in the fields of RTE, block laying, bricklaying and concreting, electrical installation welding and fabrication had the edge in some skills except for automobile vehicle repairs where female employees surprisingly held the edge with financial resource management skills. There was a significant gender difference (*p* < 0.05) in agreement to skills impacting TVET graduates’ access to employment with division along gender job descriptions.

**Discussion:**

Gender gaps exist for skills in the various disciplines of TVET in tandem with gendered job descriptions, and employability in some fields does not solely depend on the applicant’s skills. Key implications and recommendations are presented in the paper.

## 1 Introduction

Technical and vocational education and training (TVET) is a potent tool for improving livelihoods through employment (Lawrenc, 2009; von Kotze, 2008; von Kotze, 2010). Unemployment is high in Nigeria, with a remarkable impact on livelihoods. The multidimensional poverty index of Nigeria is 0.291. That puts 51.4% of the population under multidimensional poverty, with 78.4% vulnerable to poverty with low employment opportunities (UNDP, 2019). Graduates of TVET are being churned out annually from all TVET institutions in the country, with some having no hope of employment (Pathak and Emah, 2017). This contributes to greater unemployment because these institutions impart hard skills to these graduates with no provision to develop the needed soft skills or acquire them.

Academic qualification is essential to obtain decent work in the labour market. However, current trends indicate that academic qualifications alone are insufficient to obtain employment and perform well on the job (Lavy and Yadin, 2013; Succi and Canovi, 2020). Improvements in technology and the globalization have changed the demands of the labour market (Sima et al., 2020). The requirements and need for employees have changed rapidly, leading to a greater demand for contemporary trades and advanced-level skills, making it difficult for most industries to hire an appropriately skilled workforce. The consequence of this is unemployment for trained graduates (Aftab and Mohd, 2012). Soft skills training has not been mainstreamed into the curriculum of TVET institutions in Nigeria, thereby creating low employability among graduates (Awonuga, 2019; Ondieki et al., 2019). Soft skills comprise communication skills, life character traits and perspectives, people skills, career idiosyncrasy, social skills, social reasoning and emotional judgement. These skills allow people to steer through their environment, work in tandem with others, exhibit excellence in job performance and reach their targets in life with the corresponding hard skills (Jayaram and Musau, 2017). Soft skills partially determine employability, career success and achievements (Mathur, 2017).

The quality of trainees from TVET institutions in Nigeria is debatable. According to Awe et al. (2010) and Ayonmike and Okeke (2016), most graduates of technical institutions in Nigeria lack the requisite skills for employment. Meanwhile, employers demand competent TVET graduates with the requisite skills for real-world challenges (Abiodun-Oyebanji, 2014; Acheampong, 2013). Although there is a high demand for TVET skills, most graduates of TVET, especially women, have failed to gain employment due to a lack of soft skills necessary for employment (Ayonmike and Okeke, 2016). Nigerian employers have complained that TVET graduates lack the requisite skills and employability training with equivalent job performance disposition if employed (Akinloye, 2018).

Women face discrimination in career choices compared to men (Nair, 2014). The imbalance in career choice for women is the leading cause of perceived gender differences in skill levels (Kurtz-Costes et al., 2014). This difference can also be the outcome of cultural norms (Mawanga, 2016). Gaps in skill use intensity are linked to gender and other socio-economic and cultural differences (Pető and Reizer, 2021). However, the ability of women to communicate with other people in person and online is better than men (Yu, 2014).

The present study will focus on women’s and men’s self-confidence in skills and their perceived employability among TVET graduates. It aims to get a better understanding of the cause of the gender disparity in employment. The following questions guide this research:

1) Are there gender differences in hard and soft skills reported by TVET employees?
2) What are the gender differences among TVET graduates on the perceived impact of hard and soft skills on their employment opportunities?

The study makes the following two hypotheses:

*H1*: Hard and soft skills reported by employees who are TVET graduates are independent of gender.

*H2*: Perceptions of employees on the impact of hard and soft skills on TVET employment are independent of gender.

The paper is structured in the following manner: following the introduction, this paper offers a literature review on gender and TVET, covering skill acquisition and employment and ending with a list of skills used in this study. This sequence precedes the methodology, results, discussion, and conclusion sections, including recommendations.

### 1.1 Gender and technical vocational education and training

Enrolment in TVET institutions in Nigeria is unequal, with the enrolment of female students (36%) being significantly lower than male students (64%). Similarly, the percentage of male graduates (65%) was significantly higher than female graduates (35%) in 2016 (Stranger-Johannessen, 2017). For women to make a meaningful impact on Nigeria’s development process and to ensure women can reach their educational and employment potential, the participation of women in TVET needs to increase toward better gender parity.

Scholars have advanced several factors to be responsible for the low participation of women in TVET. These factors can be classified as cultural, societal, technical, and financial (Mason et al., 2012). Cultural and societal barriers that limit the participation of women in TVET include low place value on TVET in Nigerian society, which has led to a lack of recognition and discrimination against graduates of TVET (Hussaini and Jumba, 2018).

Therefore, innovations and imaginative approaches towards TVET-based employment can pave the way for gender resilience and empowerment (Foluke, 2013; Illo, 2018).

### 1.2 Gender and technical vocational education and training skill acquisition

Protracted learning concerning activities that engender specific responses due to memory-driven knowledge that leads to appropriate responses under similar circumstances can be termed “skill acquisition” (Speelman and Kirsner, 2005).

Gender norms in Nigeria tend to limit the ability of women to acquire TVET skills that would increase their chances in the labour market. According to Klugman et al. (2018), girls’ skills in an entrepreneurship programme in Nigeria were limited because parents and guardians were unwilling to prorate household chores that girls conventionally handle to other family members. In a study to examine women’s participation in skill acquisition for empowerment in Anambra state, Nigeria, Ekesionye and Okolo (2012) reported that household burden (78.1%), the influence of husbands (76.4%), and religious/cultural belief (41.9%) were top inhibitors to skill acquisition and utilisation by women. These factors hinder women’s empowerment since adult literacy and skill acquisition strongly correlate with women’s empowerment and self-reliance. The situation is similar in a neighbouring West African nation (Olagbaju, 2020). However, Danjuma et al. (2011) earlier posited that the capacity to utilise skills and not skill acquisition significantly impacts women’s empowerment in Nigeria. This contradiction stems from the fact that individual skill acquisition through training does not create the much-needed impact except when the skills are harnessed collectively through knowledge and attitude sharing within organisations. In addition, gender-specific capacity-building policies and programmes differ among African countries.

### 1.3 Employability and technical vocational education and training

Employment for TVET graduates has increased tremendously over the last few decades, with the unemployment rate among vocational and commercial education graduates going from 28.7% in 2011 to 17.9% in 2020 (ILO, 2020). This achievement is significant, considering Nigeria’s unemployment rate increased from 21.1% in 2010 to 27.1% in 2020 (ILO, 2021). However, gender-disaggregated data on unemployment shows a constant gap between men and women. In 2010, the unemployment rate in Nigeria was 17.7 and 24.9% for men and women, respectively, but it increased to 24.0 and 26.5%, respectively, in 2019, with a narrower gap (ILO, 2020). However, men’s unemployment rate in 2020 fell slightly to 22.9%, while women’s increased 5 points to 31.6%, partly because of the COVID-19 pandemic (ILO, 2020). According to Awojobi et al. (2014), women dominate employment in the manufacturing sector, accommodation, food services, human health, and social work in Nigeria. The gender gap in employability can be traced to several factors including greater access to credit and education by men, the burden of family/home care (unpaid labour) and societal gender roles (Ekesionye and Okolo, 2012).

### 1.4 Skillset and job performance

Personal attributes, achievement, and understanding are at the core of employability skills, aiming to get a job and rise successfully (Herbert et al., 2020; Mello et al., 2017). In other words, employability skills are transferable and give holders the capacity to perform work effectively (Ju et al., 2011). Attractiveness for recruitment depends on employability skills because they direct employers to adept qualifications (Cavanagh et al., 2015). This tendency creates room for job satisfaction and attainment of career peaks.

Seven verifiable employability skills relevant to achieving objectives in the industry exist. These, according to Overtoom (2000), are skills that pertain to understructure, i.e., foundation skills, which include personal attributes or quality, basic skills, and thinking skills, as well as those that pertain to proficiency in the workplace, such as technology, interpersonal, system and information skills which TVET graduates must possess regardless of gender to work with others (Mohd Jalaludin and Ihkasan, 2014; Murgor, 2017; Spinks et al., 2006). The requirement for inventiveness, docility, and task-bearing in TVET jobs demands communication skills (Bennett, 2002; Robles, 2012). This skill set is a panacea for understanding the opportunity to succeed, considering its positive effect on work performance (Sisodia and Agarwal, 2017).

Skill development and acquisition involves accumulating core entrepreneurial, communication, financial management, and leadership skills (Murgor, 2013). Core skills give job seekers an edge in job openings and self-employment (Chafa, 2015). Every TVET graduate in Nigeria may wish to become employed or become an employer of labour. This aspiration requires skills and competencies that are inherent or acquired in training. There appears to be a social construct behind core employability skills (Canning, 2006). There has been a consistent shift in terminology and semantics relating to core skills, which is related to changing cultural, political, and economic inclinations (Chafa, 2015). The historical trend of skill delivery in Africa has been that early skill acquisition focused on language skills, with a shift to enterprise skills in the 1990s.

In contrast, the current trend focuses on soft skills (Warhurst et al., 2004). The concept of skills has become very fuzzy (Warhurst et al., 2004), using various terminologies. Rasul et al. (2012) listed seven employability skills in the manufacturing industry, including thinking, information, ICT, interpersonal, basic, resource, and personal attributes. However, Chan and Fong (2018) mentioned almost completely different terminologies: ICT skills, communication skills, interpersonal skills, organisational and personal management skills, management skills, English language skills, adaptive skills, leadership skills, and problem-solving skills.

A distinction exists between hard and soft skills regarding employability and job performance. While hard skills encompass technical aspects of job performance as a direct consequence of acquiring knowledge (Chan and Fong, 2018), soft skills reflect a person’s character with a direct influence on interaction, job performance, and prospects for career development (John, 2009). It implies that hard skills are related to cognition and depend on intelligence. However, for this research, the focus is on hard and soft skills acquisition.

Graduates of TVET focuses on hard skills which is considered to be important for employment. Still, soft skills require continuous development via practical application in everyday life and the office or place of work (Robles, 2012). Considering this, graduates or employees need soft skills for better job performance. In a study of perceptions in ranking workplace proficiency between students and graduates of Business studies in New Zealand using Likert scale points, Rainsbury et al. (2002) discovered closeness of perceptions between ranking by students and graduates with regards to competencies in ICT literacy, customer care, synergy and affiliation, self-confidence, and willingness to learn, but differences existed in comparison between the two groups as regards rankings for cognitive (hard) skills and behavioural (soft) skills. Considering this, the current research identified seven soft skills and five hard skills for TVET graduates required for employability (Table 1).

**Table 1:** Hard and soft skills for employability applicable to TVET careers.

S/N	Skill	Type	Description	Source
1	Innovation skills	Soft	Skill needed to generate and apply knowledge and ideas in the workplace and in the wider society	OECD (2011) and Lowe and Marriott (2012)
2	Creative skills	Soft	Ability to make little but incremental changes to things in existence and take their application further	DeGraff and DeGraff (2020)
3	Practical skills	Hard	Aptitude for observation, handling, planning, interpreting, reporting and self-reliance	Singleton (2013)
4	Self-motivation skills	Soft	Prowess to take initiative and action towards goals and complete tasks	Marques et al. (2019)
5	Financial resource skills	Hard	Ability to manage financial systems, gather financial data, analyse financial reports and make sound financial control decisions	Paarima et al. (2021)
6	Marketing skills	Hard	Ability to anticipate needs, identify clients and satisfy them profitably	McCorkle et al. (2003)
7	Administrative skills	Hard	Targeting organisational vision by accomplishing goals, determine resources needed, and how to combine them	Cordero et al. (2004) and Hoefer (2003)
8	Entrepreneurial skills	Soft	Competence that relates to proactiveness, achievement and commitment to others	Nieuwenhuizen (2009)
9	Interpersonal skills	Soft	Etiquette and strategies for interacting with others effectively	Halfhill and Nielsen (2007)
10	Managerial skills	Soft	Capacity for executive duties while avoiding crisis situations with prompt resolution of issues	Marques et al. (2019)
11	Communication skills	Soft	Expertise in transferring information accurately, clearly and as intended	Hargie (2019)
12	Distributive skills	Hard	Ability to maintain communication, use math, troubleshoot, manage oneself, people time and products	Moore (2010)

## 2 Materials and methods

The methodology involved the administration of a questionnaire followed by quantitative analysis. The study area covered Akwa Ibom state of Nigeria. The choice of this state lies in the fact that Akwa Ibom state had the highest unemployment rate (37.7%) among all the states in Nigeria during the third quarter of 2018 (NBS, 2019).

The employee population for the study was 300 TVET graduates who are employees in six organisations that offer employment opportunities for TVET graduates in Akwa Ibom state (Table 2): (1) Broad Speed Automobile Services: Automobile repair shop (mechanical/electrical). (2) Harkata Institute of Catering/Hotel Management. (3) Major Works Limited—furniture fabrication workshop. (4) Absolute Project Global Services—welding and fabrication workshop. (5) Seal World Technology Services: ICT training and computer/electronics repair shop. (6) Ken Johnson Construction Services: building construction and renovation contractors (who are recruiting TVET graduates as permanent employees). These organisations were selected because they are the largest employers of TVET graduates in the state. The target population (sample size) for this study resulted from a 5% level of precision on the total population using Yamane’s formula (Yamane, 1967):

$$n=\frac{N}{1+N{\left(e\right)}^{2}}$$

(where *n* = sample size, *N* = population size and *e* = level of precision).

**Table 2:** Distribution of the study population of employees who are TVET graduates and study sample.

Organisations	Total population	Sample size	Female	Male
Broad speed Automobile Services	48	28	14	14
Harkata Institute of Catering/Hotel Management	54	30	15	15
Major Works Limited	45	28	14	14
Absolute Project Global Services	48	28	14	14
Seal World Technology Services	51	28	14	14
Ken Johnson Construction Services	54	30	15	15
Total	300	172	86	86

The respondents were employees of the organisations with working experience ranging between 1 month and 24 months.

Upon determining the total sample size, each organisation’s sample size originated from a simple percentage of the total population drawn from the total sample size. Approximations were made to the sample sizes in each organisation to obtain even sample sizes that can be divided equally between both genders—the people administered with questionnaires comprised of respondents drawn using a gender disaggregated list of employees.

### 2.1 Survey of employees who are TVET graduates

The respondents were selected by systematic random sampling using the TVET employees list from the six organisations. The selection of respondents involved every second staff on the gender-disaggregated staff list in each organisation until the target sample for each gender was met. Hence a total of 86 respondents were selected per gender. Administration of the questionnaire involved a single questionnaire for each selected employee. The questionnaire consisted of 55 items, divided into five sections: personal information, factors influencing men and women in TVET skill acquisition, gender barriers to training/employment opportunities, perception of the possession of the previously listed five hard skills and seven soft skills, and gender expectations and difficulties in meeting employment requirement. The respondents were given the list of the skills and the corresponding description (Table 1) to enable them to digest the meaning of each skill and determine their level of confidence in each skill before ticking the skills they perceive they have.

Descriptive (percentages and frequency counts, bar graphs, and arithmetic means) items and parametric (two sample *z* test, *t*-test, *χ*^2^ test and correlation) tests revealed trends in the data. Data were analysed using IBM^®^ SPSS Statistics 21 software (George and Mallery, 2019; IBM, 2012) and R v 3.4.3 (R Core Team, 2017).

## 3 Results

### 3.1 TVET courses studied by respondent employees

**Figure 1:**
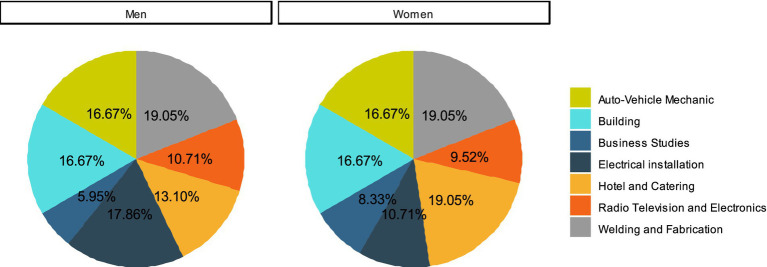
Gender disaggregated data of courses taken by employees who are TVET graduates.

The distribution of respondents who are employees who are TVET graduates according to courses they studied (Figure 1) reveals that courses taken were independent of gender (*χ*^2^ = 2.82; df = 6 *p* = 0.831). The percentage of female and male respondents was equal in four out of seven fields, including welding and fabrication, auto-vehicle repairs, building and business studies.

### 3.2 Skills reported by employees

#### 3.2.1 TVET field: business studies

In the area of business studies, there was a difference in skill confidence (*p* < 0.05) in favour of female employees in five skills compared to male advantage in just one skill (Table 3). Women expressed more confidence in the soft skills of innovation, interpersonal relations and management than men. This confidence agrees with an earlier report that women display better competence in innovation, interpersonal communication and office politics than men (Poole et al., 1998). Confidence in self-motivation skills, a soft skill, as perceived by men, is a common occurrence in the business world, where men tend to be over-confident (Doldor et al., 2021). There was gender parity in confidence for creative skills, financial resource skills, administrative skills, entrepreneurial skills, communication skills, and distributive skills (*p* > 0.05). While both genders generally exhibit confidence in skills in their rights, women tend to be more collaborative and effective communicators. This attribute is beneficial in an entrepreneurial and creative environment like the business world (Guillen, 2018). Female employees do not report confidence in self-motivation skills, while employees who are men lack interpersonal, managerial, and marketing skills. These results show a gender difference in confidence for skills among employees who are TVET graduates of business studies, in Akwa Ibom state.

**Table 3:** Confidence in skills reported by employees who are graduates of business studies.

Confidence	Employees (%)		*p*-value	Difference	Skills
	Female	Male			
Innovation skills	42	25	0.049	Female	Soft
Creative skills	25	25	1.000	—	Soft
Practical skills	58	25	3.78 × 10^−04^	Female	Hard
Self motivation skills	0	17	1.53 × 10^−05^	Male	Soft
Financial resource skills	8	8	1.000	—	Hard
Marketing skills	25	0	5.96 × 10^−08^	Female	Hard
Administrative skills	17	8	0.108	—	Hard
Entrepreneurial skills	25	17	0.280	—	Soft
Interpersonal skills	25	0	5.96 × 10^−08^	Female	Soft
Managerial skills	17	0	1.53 × 10^−05^	Female	Soft
Communication skills	17	8	0.108	—	Soft
Distributive skills	8	8	1.000	—	Hard

#### 3.2.2 TVET field: radio, television, and electronics repair

The confidence in skills reported by graduates/employees in the field of radio, television, and electronics (RTE) repair (Table 4) shows men scored significantly higher (*p* < 0.05) in three skills: creative skills, self-motivation skills, and managerial skills. This difference suggests that gender-based differences may exist in how TVET training in RTE repair is perceived and experienced, with men feeling more confident in three soft skills than their female counterparts. This higher level of confidence in creativity by the men disagrees with the record that women score better in creativity (Baer and Kaufman, 2008). These findings may have important implications for how TVET programs are designed and delivered, with a need to ensure that all trainees, regardless of gender, have access to the support and resources they need to develop their skills and succeed in their chosen careers. There was no statistically significant difference (*p* > 0.05) for reported confidence in nine other skills. The confidence in entrepreneurial skills and innovation skills rank high among female employees (35% respectively). In contrast, confidence in practical, creative and financial resource skills rank next in that order. These findings suggest that female employees possess strong entrepreneurial and innovation skills. These skills can benefit their organisations. Employers need to recognize and support these strengths in their female employees, as they may contribute significantly to the success and growth of the business. Additionally, the results highlight the need for training and resources to develop practical, creative, and financial resource skills among female employees to enhance their confidence levels further. Among the male employees, confidence in two skills were common: innovation skills and creative skills, with 41% occurrence, respectively. Women did not report confidence in managerial skills. These results suggest that there may be gender-based differences in the types of skills that are emphasized or valued in TVET training programs. Reports show that male trainees are encouraged or supported in developing their innovation and creative skills, while female trainees may not receive the same level of attention or resources in developing their managerial skills (Klugman et al., 2018; Ekesionye and Okolo, 2012). Traditional gender stereotypes equate men with innovation and creativity and women with communication skills. Thus, men in this area of TVET receive more support than women. As a result of this stereotype, men are more likely to be encouraged to work in STEM (Science, Technology, Engineering, and Mathematics) sectors, while women are pushed toward careers that need social intelligence. Women in RTE often lack confidence in their abilities since they cannot find female role models in the industry.

**Table 4:** Confidence in skills reported by employees who are graduates of radio, television, and electronics repair.

Confidence	Employees (%)		*p*-value	Difference	Skills
	Female	Male			
Innovation skills	35	41	0.567	—	Soft
Creative skills	24	41	0.046	Male	Soft
Practical skills	29	35	0.532	—	Hard
Self motivation skills	12	29	0.012	Male	Soft
Financial resource skills	24	12	0.065	—	Hard
Marketing skills	12	6	0.238	—	Hard
Administrative skills	6	12	0.238	—	Hard
Entrepreneurial skills	35	35	1.000	—	Soft
Interpersonal skills	12	24	0.065	—	Soft
Managerial skills	0	12	4.88 × 10^−04^	Male	Soft
Communication skills	18	29	0.144	—	Soft
Distributive skills	6	12	0.238	—	Hard

#### 3.2.3 TVET field: automobile vehicle repairs

Women score higher (*p* < 0.05) regarding confidence in financial resource skills (18%) among employees who are TVET graduates of automobile vehicle repairs (Table 5) than men (0%). There was gender parity (*p* > 0.05) for confidence in all the other skills. This gender similarity in skill confidence for other skills suggests that the gender difference in confidence in financial resource skills may be unique to this skill set. It is also worth noting that broader societal factors, such as gender norms and expectations, may influence the gender difference in confidence in financial resource skills considering, the role of women as home managers (Kamath and Dattasharma, 2015). Financial resource skill is a hard skill acquired in the TVET programmes through entrepreneurship education. Therefore, an understanding of the principles of financial resource management begets competence. The delivery of entrepreneurship education in TVET strongly influences skills in financial resource management (Padi et al., 2022).

**Table 5:** Confidence in skills reported by employees who are graduates of automobile vehicle mechanic works.

Confidence	Employees (%)		*p*-value	Difference	Skills
	Female	Male			
Innovation skills	29	36	0.457	—	Soft
Creative skills	32	21	0.169	—	Soft
Practical skills	29	32	0.798	—	Hard
Self motivation skills	25	21	0.659	—	Soft
Financial resource skills	18	0	7.63 × 10^−06^	Female	Hard
Marketing skills	11	11	1.000	—	Hard
Administrative skills	18	14	0.597	—	Hard
Entrepreneurial skills	32	21	0.169	—	Soft
Interpersonal skills	18	11	0.265	—	Soft
Managerial skills	21	25	0.659	—	Soft
Communication skills	29	21	0.322	—	Soft
Distributive skills	14	11	0.690	—	Hard

#### 3.2.4 TVET field: block laying, bricklaying, and concreting (building)

The percentage of male employees who are graduates of block laying, bricklaying, and concreting (Table 6) is greater than the female employees reporting confidence in two skills: practical skills (43%:21%) and financial resource skills (29%:14%). The gender difference in practical skills is significant and suggests that male graduates are twice as confident in their practical and financial resource skills than female graduates. This difference may be due to social and cultural factors discouraging women from pursuing careers in traditionally male-dominated fields such as construction. More so, employers in Africa now demand practical skills (Aboagye and Puoza, 2021) and this places the women that are trained in the field of block laying, bricklaying, and concreting at a disadvantage. The implication here is that men are twice as likely to get the jobs than women. Regarding financial resource skills, the confidence of men compared to women has been linked to the belief and stereotype that women are less knowledgeable in construction skills despite the fact that they are successful home builders and administrators (Maharaj and Edigheji, 1999). However, it is essential to note that there was gender parity (*p* > 0.05) in perceived confidence levels for the other skills among the respondents. This gender parity suggests that gender differences in confidence levels are not universal and may be specific to certain skills or domains.

**Table 6:** Confidence in skills reported by employees who are graduates of block laying, bricklaying, and concreting.

Confidence	Employees (%)		*p*-value	Difference	Skills
	Female	Male			
Innovation skills	46	43	0.832	—	Soft
Creative skills	25	39	0.103	—	Soft
Practical skills	21	43	0.008	Male	Hard
Self motivation skills	25	21	0.659	—	Soft
Financial resource skills	14	29	0.032	Male	Hard
Marketing skills	18	18	1.000	—	Hard
Administrative skills	7	14	0.189	—	Hard
Entrepreneurial skills	32	32	1.000	—	Soft
Interpersonal skills	11	14	0.690	—	Soft
Managerial skills	14	21	0.311	—	Soft
Communication skills	18	25	0.360	—	Soft
Distributive skills	18	11	0.265	—	Hard

#### 3.2.5 TVET field: electrical installation

There was a gender difference (*p* < 0.05) in reported confidence in practical skills, with more men reporting confidence in practical skills (Table 7). This finding suggests that social and cultural factors may influence the confidence levels of men and women in practical skills. Men may be more likely to receive encouragement and support in pursuing practical skills, while women may face more barriers and stereotypes that discourage them from pursuing these skills. Additionally, women may have less access to training and education in practical skills in this field due to male-centred delivery methods, which could further contribute to lower confidence levels. However, it is essential to note that there was no gender difference (*p* > 0.05) in reported confidence levels for all other skills among the respondents. This gender similarity in skills suggests that gender differences in confidence levels are specific to practical skills.

**Table 7:** Confidence in skills of employees who are graduates of electrical installation.

Confidence	Employees (%)		*p*-value	Difference	Skills
	Female	Male			
Innovation skills	25	29	0.683	—	Soft
Creative skills	21	25	0.659	—	Soft
Practical skills	17	42	0.002	Male	Hard
Self motivation skills	25	33	0.358	—	Soft
Financial resource skills	17	8	0.108	—	Hard
Marketing skills	17	8	0.108	—	Hard
Administrative skills	17	17	1.000	—	Hard
Entrepreneurial skills	25	33	0.358	—	Soft
Interpersonal skills	21	13	0.229	—	Soft
Managerial skills	17	21	0.627	—	Soft
Communication skills	17	13	0.585	—	Soft
Distributive skills	17	13	0.585	—	Hard

#### 3.2.6 TVET field: hotel and catering

The results of Table 8 indicate a significant gender difference (*p* < 0.05) in the skill confidence levels of employees with hotel management and catering training. This difference is in favour of female employees. Moreover, the percentage of women employed in this sector of the economy is higher than that of men, with women accounting for 86.56% of the workforce (NBS, 2017). Interestingly, women in this industry reported confidence in all (hard and soft) skills. This confidence level suggests that women are skilled in practical aspects of the job and excel in interpersonal skills, such as communication and teamwork. This finding challenges traditional gender stereotypes that associate women with soft skills and men with hard skills. The ability of women to perform better than men in the hospitality industry has been shown by Baum (2013) with survey responses that show that 82% of respondents feel women should not be confined to unskilled positions in the hospitality industry. The research also showed that 79% of respondents feel that the presence of women within the workforce in the hospitality industry creates an overall improvement in the quality of the workforce. The stereotype that men are associated with hard skills seems to operate only in male dominated fields and this is the reason why Torre (2019) reports that only 25% of men previously employed in female dominated fields would return to jobs in that field.

**Table 8:** Confidence in skills of employees who are graduates of hotel management and catering.

Confidence	Employees (%)		*p*-value	Difference	Skills
	Female	Male			
Innovation skills	54	29	0.008	Female	Soft
Creative skills	50	14	7.07 × 10^−06^	Female	Soft
Practical skills	54	18	2.57 × 10^−05^	Female	Hard
Self motivation skills	46	18	0.001	Female	Soft
Financial resource skills	39	4	3.11 × 10^−08^	Female	Hard
Marketing skills	50	14	7.07 × 10^−06^	Female	Hard
Administrative skills	39	14	0.001	Female	Hard
Entrepreneurial skills	46	18	0.001	Female	Soft
Interpersonal skills	36	18	0.020	Female	Soft
Managerial skills	50	29	0.024	Female	Soft
Communication skills	43	21	0.008	Female	Soft
Distributive skills	46	7	4.00 × 10^−08^	Female	Hard

#### 3.2.7 TVET field: welding and fabrication

The percentage of men with confidence in interpersonal and communication skills among employees who are graduates of welding and fabrication (Table 9) is significantly higher than that of women (*p* < 0.05). Gender disparities in social influence and the techniques employed to exert influence reflect these distinctions to varying degrees (Torppa, 2010), especially when we consider the fact that men dominate the welding and fabrication field. In most situations, women have a harder time influencing others than men have, especially when persuading others of their expertise or ability (Danjuma et al., 2011). These results suggest that gendered job roles play a mediating role in the influence gap between the sexes. There was no statistically significant (*p* > 0.05) gender difference in confidence for all the other skills among the employees in this field. This parity suggests that, for the most part, men and women in this field may share similar levels of confidence beyond interpersonal and communication skills. Men have been shown to rate instrumentally oriented communication skills over affectively oriented communication skills preferred by the women (Burleson et al., 1996). This therefore means that the confidence in interpersonal and communication skills by men is directly linked to the fact that this field utilises instruments for work.

**Table 9:** Confidence in skills of employees who are graduates of welding and fabrication.

Confidence	Employees (%)		*p*-value	Difference	Skills
	Female	Male			
Innovation skills	34	34	1	—	Soft
Creative skills	38	34	0.724	—	Soft
Practical skills	25	25	1	—	Hard
Self motivation skills	16	22	0.418	—	Soft
Financial resource skills	16	25	0.211	—	Hard
Marketing skills	13	25	0.073	—	Hard
Administrative skills	9	19	0.087	—	Hard
Entrepreneurial skills	34	28	0.526	—	Soft
Interpersonal skills	9	25	0.009	Male	Soft
Managerial skills	19	28	0.243	—	Soft
Communication skills	16	31	0.04	Male	Soft
Distributive skills	13	19	0.377	—	Hard

### 3.3 Overall confidence in skills

Considering the skills without regard to the field of work, there was generally no difference in proportions of women and men in their perception of confidence in the soft and hard skills (Table 10). The proportion of women who expressed confidence in entrepreneurial skills was greater than that of the men (*p* < 0.05).

**Table 10:** Confidence in soft and hard skills of employees of TVET in Akwa Ibom state.

Skills	Proportion		*t*-statistic	*p*-value
	Female	Male		
Soft skills				
Innovation skills	0.523	0.477	1.060	0.310
Creative skills	0.518	0.482	0.490	0.633
Self motivation skills	0.421	0.579	−1.287	0.222
Entrepreneurial skills	0.557	0.443	2.275	0.042^*^
Interpersonal skills	0.563	0.437	0.960	0.356
Managerial skills	0.477	0.523	−0.284	0.781
Communication skills	0.520	0.480	0.541	0.599
Hard skills				
Practical skills	0.499	0.501	−0.020	0.985
Financial resource skills	0.639	0.362	2.048	0.063
Marketing skills	0.639	0.361	2.401	0.033^*^
Administrative skills	0.495	0.505	−0.104	0.919
Distributive skills	0.551	0.449	1.106	0.291

However, when looking at this from the angle of the fields of study, findings show that women have an edge in perceived confidence in entrepreneurial skills only in the field of hotel management and catering with equal confidence levels with the men in all the other fields.

Among the hard skills, there was a gender difference in the confidence levels for the skill of marketing. There was a significantly greater proportion (*p* < 0.05) of women who see themselves as confident in this skill compared to men. This difference can be explained by the fact that women had higher confidence levels in this skill within two fields: business studies and hotel management and catering.

### 3.4 Impact of reported skills on access to employment

Access to employment as a result of the skills respondents reported that they possess (Table 11) shows that there was a gender difference (*p* < 0.05), with more men agreeing that their skills in business studies (41.7%) and automobile vehicle mechanics (42.9%) influenced access to employment while more women disagree that their skills in these two fields influenced access to employment (16.7 and 17.9% respectively). The gender difference in the perceived influence of skills on access to employment may be due to various factors, such as gender stereotypes and biases in the job market (Ekesionye and Okolo, 2012). A recent report corroborates this finding with a statistic showing only 62.5% of working-age women were employed, whereas 75.9% of men were (World Bank, 2022). In addition, the percentage of women in the field of business studies that remain undecided (16.7%) was significantly (*p* < 0.05) higher than the percentage of men (0.0%). This indecision by women in business studies suggests they may be less confident in their skills or face more barriers in accessing employment opportunities than men. The implementation of women in development intervention in the 1990s overlooked this situation (Njiro, 1999). There was a gender difference (*p* < 0.05) with more men uncertain on whether their skills in electrical installation or their soft skills give them access to employment (16.7% of men vs. 4.2% of women). The indecision in this case, may be caused by the low place value of TVET in Nigeria (Hussaini and Jumba, 2018). In contrast, there was a gender difference (*p* < 0.05) with more women agreeing that their soft skills and hard skills in hotel management and catering impact their access to employment (28.6% of women vs. 14.3% of men). This finding suggests that women in hotel management and catering emphasise soft and hard skills as critical factors for accessing employment, a situation already demonstrated by Sisson and Adams (2013) for soft skills and Hight et al. (2019) for hard skills. In the fields of RTE, block laying, bricklaying/concreting, and welding and fabrication, there was no gender difference (*p* > 0.05) in the impact of skills on their access to employment. The percentage agreement to the impact of their skills on employment was ≥25.0% in each case. These statistics suggest that, among the employees of these fields mentioned above, there is no evidence of a gender difference in the perceived impact of skills on employment. However, other factors, such as experience and access to information, could impact employment outcomes in these fields, regardless of gender (Okoye and Edokpolor, 2021).

**Table 11:** Employees’ perception regarding the influence of their reported skills on their access to employment.

TVET field	Agree (%)			Disagree (%)			Undecided (%)		
	Female	Male	*p*-value	Female	Male	*p*-value	Female	Male	*p*-value
Business studies	25.0	41.7	0.049^*^	16.7	0.0	1.52 × 10^–05*^	16.7	0.0	1.52 × 10^−05*^
RTE	29.4	35.3	0.620	11.8	11.8	1.000	5.9	5.9	1.000
Automobile vehicle mechanic	25.0	42.9	0.038^*^	17.9	0.0	7.63 × 10^−06*^	7.1	7.1	1.000
Block laying, bricklaying and concreting	28.6	35.7	0.382	10.7	7.1	0.481	10.7	7.1	0.481
Electrical installation	25.0	37.5	0.130	8.3	8.3	1.000	4.2	16.7	0.007^*^
Hotel management and catering	28.6	14.3	0.032^*^	14.3	21.4	0.405	14.3	7.1	0.189
Welding and fabrication	37.5	25.0	0.130	9.4	15.6	0.230	3.1	9.4	0.267

Key: ^*^Statistical significance (*p* < 0.05).

The correlation between the confidence of men in their skills and their perception on employability is negative and very low (*r* = −0.15) but not significant (*p* = 0.740). There was a very low correlation (*r* = −0.013) between the confidence in skills of the women and their perception on employability. This relationship is negative and not significant (*p* = 0.980). These suggest that there is no significant linear relationship between the skill confidence level of women and their perception of employability. This phenomenon is similar to the negative correlation between self-esteem and competence on the job as identified in nurses by Serafin et al. (2022), but the result in this case was significant. Looking at the correlations between women and men, there was a strong negative correlation (*r* = −0.590) between the perception of women on the impact of their skills on employment and that of the men. This correlation is not significant (*p* = 0.160). The implication of this finding is that a population-wide estimation of the relationship between gender perceptions on the impact of skills on employment is only possible with a larger number of respondents. There is a strong positive correlation (*r* = 0.630) between the confidence of men in their skills and the perception of women on their employability given their own skills. The relationship is however not significant (*p* = 0.130). There is a strong and significant (*p* = 0.050) negative correlation (*r* = −0.790) between the confidence level of women in their skills and the perception of the men on their own employability given their skills. The interpretation of this statistic is that either the women’s confidence in skills will increase and the perception of men that their skills grant them employment will decrease or vice versa. This suggests that when women’s confidence in their skills increases, the perception of men in their skills as a channel for employment will decline even in male-dominated fields. The confidence of men in their skills and that of the women have a negative correlation (*r* = −0.40), and it is not significant (*p* = 0.370). This signifies a lack of linear relationship between the variables.

## 4 Discussion

A deconstruction of gaps and similarities in confidence in skills reported by respondents in this study reveals some interesting details. There is gender difference in confidence in skills according to TVET fields that conforms to Nigerian society’s socio-cultural beliefs, which define gender roles. This stereotype diminishes the capacity of women to use skills they acquire in training or have confidence in Danjuma et al. (2011).

The findings revealed that women employed in TVET fields that men dominate lack confidence in some soft and hard skills. Women in RTE and welding and fabrication lack confidence in some soft skills. To understand why this situation exists among women in the field of RTE, we first need to know that gender roles in society have placed barriers to entrepreneurship and financial independence for women (Mason et al., 2012; Hussaini and Jumba, 2018). These barriers may have hindered women’s skills development and impacted their confidence in their abilities to perform well in these fields. Women have unique needs and work-life balance requirements not met in these male-dominated fields (Martin and Barnard, 2013), which could hamper their ability to deliver soft skills. In RTE, women reported less confidence in creativity, self-motivation and managerial skills. These soft skills can be honed on the job. This possibility notwithstanding, self-perception, as opposed to the prevailing circumstance in the workplace, is a determinant of motivation and subsequent achievement (Nasution et al., 2014). This research indicates that although the assumption that gender influences career choice was not found in this study, it influenced women’s self-perception on the job. It undermines the ability of women in the TVET field of RTE to express their creative and managerial capabilities and their self-motivation on the job. In welding and fabrication, there is a gender gap in confidence in two soft skills: interpersonal and communication skills in favour of the men. Women use expressions and nonverbal communication more than men (Briton and Hall, 1995). This difference in approach to communication can hamper the utilisation of their communication skills within a male-dominated environment. Research has shown that the lack of confidence by women in male-dominated work environments affects their level of confidence. This has implications on work performance and achievement (Okoye and Edokpolor, 2021), considering that women’s opinions are often overlooked (Dorrance Hall and Gettings, 2020). Therefore, the finding in this research that shows women have a confidence gap in interpersonal skills in male-dominated field of welding and fabrication is not strange.

In this study, women in the fields of automobile vehicle repair, bricklaying and concreting and electrical installation expressed less confidence in two hard skills: practical and financial resource skills. The low confidence in hard skills depend on the quality of training (Awe et al., 2010; Ayonmike and Okeke, 2016) and the influence of gender and relationships (Pető and Reizer, 2021). Women’s level of confidence in skills is impacted by their acceptance within male-dominated fields (Kurtz-Costes et al., 2014) and society’s disposition (Mawanga, 2016). The paucity of confidence in hard skills in women that have chosen these male-dominated fields can be overcome by changes in the TVET curricula since the training needs of women are quite often different from that of men (Jutting and Morrisson, 2009).

Perception of access to employment due to confidence in skills among employees who are TVET graduates is rooted in societal job roles. Female employees believe their skills propel them to access jobs in hotel management, a female-dominated field in the country. The perception that female employees’ skills propel them towards jobs in hotel management may be rooted in the societal expectation that women are more suited to certain jobs. In the present study, the women employed in this field clearly expressed greater confidence in their skills than the men. This consciousness in the women was responsible for their rating the skills as stepping stones to their employment in hotel management and catering. This present result corroborates the report by Awojobi et al. (2014) on the dominance of women in the field of hospitality.

Although women with TVET training in Akwa Ibom state employed in the field of business studies expressed confidence in more skills than the men, there was a gender difference in perception that these skills led them to employment. A greater proportion of the study population who were men (41.7%) perceived that their skills were responsible for their access to employment as against 25% of responses from the women. Interestingly, there was a gender difference in response regarding disagreement with skills being responsible for access to employment. This disagreement was expressed by the women (16.7%) and not the men (0.0%). In addition, the same percentage of responses were recorded for women who remained undecided concerning the perception that their skills granted them access to employment. These findings suggest that there are gender differences in how individuals perceive the role of skills in accessing employment, and these differences may be influenced by societal norms and expectations. The findings also show that women in business studies reported greater confidence than the men in the soft skills of innovation, interpersonal relations and managerial capacity and in two hard skills: practical and marketing skills. However, the women did not feel confident that their skills were being considered in granting employment. The results from this study have shown a very weak negative relationship between the perception of women on their employability and their confidence in skills. The practical explanation therefore is that the confidence of women in their skills is being affected by other factors. Men have a high level of ego within the business world. The women are not being taken seriously as reported by Woldie and Adersua (2004). The dissatisfaction of women in employment under business studies concerning this marginalization is expressed in their disagreement concerning the use of their skills in granting them employment. Moreover, the same proportion of women remain undecided concerning the role of their skills in accessing employment. This clearly shows that the societal gender roles and expectations have made them oblivious to their marginalization as shown earlier by Clegg and Mayfield (1999).

In the field of automobile vehicle repairs, the proportion of women with confidence in financial resource skills was greater than that of men. Our findings showed that the men tended to believe their skills were responsible for obtaining employment, while a greater proportion of the women disagreed with this. The contradiction in financial resource skill confidence between women in the automobile repair field and women in the block and bricklaying field needs further insight. However, it is important to state here that women in the automobile repair field can interact with other women who bring cars for repair while those in the block and bricklaying field work mostly with men who are the contractors, engineers and foremen at construction sites. This lack of interaction with peers could lead to depressed confidence in skills.

Although there was no gender difference in perception that skills granted the respondents access to employment in the field of electrical installation, there was a gender difference in the proportion of respondents that could not decide in favour or against their skills as steppingstones to their employment. A greater proportion of the men (16.7%) were not sure if their skills granted them access to employment. This gender difference in uncertainty about the role of skills in employment opportunities in the field of electrical installation is a noteworthy finding. It suggests that men may have more doubt or hesitation about the connection between their skills and employment opportunities than women. This finding negates the narrative in the literature on masculine dominance in this field alongside societal gender expectations of male confidence. Further research is needed to determine the reasons for this gender difference and to identify any underlying barriers that may be preventing men from fully recognizing and utilizing their skills in this field.

It has been observed that there could be differences in employment outcomes across majors, as fields of study signal different types of human capital (Lavy and Yadin, 2013). This indicates that the impact of skills on employment may vary depending on the specific field of study and the associated human capital.

The influence of reported skills on employment access in the field of business studies varies between genders. A greater percentage of men agree that reported skills influence their access to employment, while a significant percentage of women disagree or remain undecided on this matter. This finding aligns with existing research that highlights gender disparities in various aspects of business and entrepreneurship. For instance, studies have shown that there are structural dissimilarities between male-owned and female-owned businesses, which explains most of the contrasting funding profiles (Lawrenc, 2009). Additionally, research has indicated that there is a significant underperformance in the size, growth, and efficiency of firms owned by women when compared to those owned by men (von Kotze, 2008). These disparities in business performance and access to credit may contribute to the differing perceptions of the influence of reported skills on employment access between genders in the field of business studies.

Moreover, the influence of gender stereotypes and societal expectations on career aspirations and choices cannot be overlooked. Research has demonstrated that the attribution of masculinity to certain subjects, including those related to business and entrepreneurship, does not differ significantly among female students, indicating the presence of gender stereotypes that may influence career preferences and beliefs about employment access (von Kotze, 2010). Furthermore, the study on the characteristics influencing willingness to invest in female-versus male-led start-up companies in STEM and non-STEM fields revealed demographic differences that may explain variation in results, pointing to the complex interplay between gender, perception of success, and investment decisions in business contexts (UNDP, 2019). The gender disparities in business performance, access to finance, and career outcomes are multifaceted and influenced by a range of factors, including societal norms, structural differences, and investment biases (Kamath and Dattasharma, 2015).

Although gender is categorical, Ugwoke et al. (2021) reported that the physical demands and risks associated with radio and television technicians are not gender-specific. Additionally, the need analysis for infusing entrepreneurship skills into the radio, television, and electronic work programs in technical colleges in North-Western Nigeria emphasized the importance of enhancing skills across the curriculum, irrespective of gender, to improve employability (Shuaibu et al., 2019).

The impact of skills on employability in the TVET field of automobile vehicle repairs shows a gender disparity. A higher percentage of men agreed that their skills contribute to their employment prospects, while a higher percentage of women disagreed compared to men. This gender-based difference highlights the potential for unequal opportunities and challenges faced by women in this field. The equal percentage of undecided respondents from both women and men suggests a need for further research to understand the factors contributing to this uncertainty.

This gender disparity in the response to the impact of skills on employability aligns with empirical studies that have confirmed disparities at both macro and micro levels (Ewuoso, 2024). Furthermore, the imbalance between labor supply and demand, as well as regional disparities in labor resources, may contribute to the observed gender differences in the TVET field of automobile vehicle repairs (Vasyl’yeva et al., 2023). These disparities may be influenced by broader societal and economic factors, which could impact the opportunities available to individuals based on their gender (Kamath and Dattasharma, 2015).

The perception of skills as a stepping stone for employment in the TVET field of block laying, bricklaying, and concreting was found to be unrelated to gender. This suggests that gender did not play a significant role in shaping the perceptions of employability in this specific field. This finding aligns with the notion that in certain vocational and technical fields, gender may not be a determining factor in the perception of skills and their relationship to employment opportunities (Brockington and Cicmil, 2016). It also underscores the importance of recognizing the diversity of experiences and perspectives within different vocational and technical domains.

This gender difference in undecidedness may be influenced by various factors, including social expectations (Kamath and Dattasharma, 2015), confidence levels (Awe et al., 2010; Ayonmike and Okeke, 2016), or specific experiences within the field (Martin and Barnard, 2013). This is particularly true since there was a gender difference recorded for practical skills, with men reporting more confidence than women.

The impact of skills on employment of TVET graduates in the field of hotel management and catering in Akwa Ibom state exhibited a gender difference in agreement, while there was no effect of gender on the perception of disagreement and undecidedness. This suggests that gender may influence how individuals perceive the relationship between their skills and employability in this specific TVET field.

The gender difference in agreement aligns with research that has documented gender disparities in perceptions of employability and career success in various contexts (Park et al., 2016). Understanding the relationships between gender discrimination, career success, and human resource management can provide insights into the factors contributing to the gender difference in agreement in hotel management and catering (Park et al., 2016). Additionally, the level of implementation of the lifelong learning program in the catering and serving cluster has been shown to have a high impact on skill improvement and income generation, which may also influence perceptions of employability in this field (Lidon et al., 2022).

The absence of a gender effect on the report of disagreement and undecidedness suggests that while men and women may agree or disagree differently on the impact of skills on employment, their uncertainty about this relationship is not influenced by gender. This finding underscores the need to explore the specific factors that contribute to the gender difference in agreement within the field of hotel management and catering. For instance, the gap in soft skills perceptions and the challenges in manager-employee cooperation may shed light on the complexities of gender dynamics in perceptions of employability within this field (Chytiri et al., 2020).

Innovation has been the guiding force for the development of employment opportunities for the technical and vocational education and training (TVET) graduates. However, in Nigeria, it has been found that there is a lack of infrastructure and policy implications for the inclusion of innovative technologies in TVET (Adeagbo et al., 2024). There is a dire need to improve the policy level, curriculum development and government interventions towards strengthening the innovative, creative means of generating sustainable entrepreneurship (Eno Obot Jackson et al., 2022). Therefore, despite the positive interventions of the Artificial Intelligence technological advancements, TVET in Nigerian context has to be strengthened at all levels of governance (Omeh et al., 2024).

The data collection for this research highlighted several skills deemed necessary for the TVET graduates in Nigeria. The top five skills observed during the data collection are as follows:

- Detailed perspectives towards finding solutions to the real work life issues. One of the respondents added that the upholstery industry requires precision levels from the TVET graduates.
- Adaptability and problem solving skills were among the top skills required. The ever changing business, technical and gender scenarios drive the TVET graduates to be equipped with this skill. The data was found among the respondents in the event management sector.
- Communication: Another top skill was observed to be good communication skills. This was found to be one of the essential skills required by all the employees in the study area.
- Planning and time management was found to be among the top five skills among the TVET graduates. With the hectic schedules, deadlines and focus on the quality based services of TVET graduates, it was found to be essential to have this skill.
- Innovation, creativities and imagination was found to be the fifth top skill required among the TVET graduates in Nigeria. The inclusion of AI could be an opportunity among the TVET graduates in future.

Therefore, gender based further research is required in context of AI and inculcating the employment opportunities in Nigeria.

The findings are consistent with the importance of employability skills for TVET graduates, regardless of gender, as highlighted in the study on appraising fabrication and welding students’ employability skills in Ogun state technical colleges (Olawale and Olaseni, 2019). Additionally, the emphasis on employability skills in TVET underscores the necessity for all graduates to possess these skills, irrespective of gender, as indicated in the study on employability skills in TVET (Nugraha et al., 2020; Griffiths et al., 2018; De Weert, 2007; Clarke, 2018). These findings are crucial for promoting gender equality and inclusivity in the TVET field of welding and fabrication, emphasizing the importance of skills and competencies for all graduates, regardless of gender.

## 5 Conclusion

This study examined the soft and hard skill perception of TVET-trained men and women currently employed within the private sector in Akwa Ibom state and the impact of confidence in skills on their employment. Reports of confidence in soft and hard skills and reported impact of confidence in skills on employment were dependent on gender in four TVET fields. Gender stereotypes shaped the responses to the effect of confidence in skills on employment.

The findings underscore the importance of addressing gender disparities in TVET fields. Efforts to promote gender equality and provide equal opportunities for skill development and employment are crucial. Additionally, understanding the factors that contribute to the differing perceptions of employability based on skills and gender is essential for developing targeted interventions to address these disparities.

Efforts to address the gender difference in agreement and to understand the factors contributing to this disparity are crucial for promoting gender equality and inclusivity in the TVET field of hotel management and catering. Policymakers and educators must come together to formulating TVET educational policies and regulations. This is essential for adequate policy formulation as well as the accurate implementations of the policies thereof. Furthermore, considering the perceptions of employability and the development of skills in this field is essential for enhancing the career prospects of both men and women in Akwa Ibom state.

## Data Availability

The original contributions presented in the study are included in the article/supplementary material, further inquiries can be directed to the corresponding author.
